# Extent and Association of Smartphone Usage With Musculoskeletal Outcomes Among Medical Students: A Comparative Cross-Sectional Study

**DOI:** 10.7759/cureus.108199

**Published:** 2026-05-03

**Authors:** Priyadharshini Nattalam Adikesavan, Rajprasath Ramakrishnan, Dinesh Kumar V, Padmanabhan Elamparidhi

**Affiliations:** 1 Anatomy, Sri Manakula Vinayagar Medical College and Hospital, Puducherry, IND; 2 Anatomy, All India Institute of Medical Sciences, Madurai, Madurai, IND; 3 Anatomy, Jawaharlal Institute of Postgraduate Medical Education and Research, Puducherry, IND; 4 Radiodiagnosis, Sri Manakula Vinayagar Medical College and Hospital, Puducherry, IND

**Keywords:** cranio-vertebral angle, neck pain, posture, smartphone addiction, ultrasonography

## Abstract

Introduction

As gadgets become a part of human life, smartphone (SP) use involves repetitive hand and digit movements that may contribute to musculoskeletal strain, especially in the thumb, neck muscles, back muscles, and upper limb joints. Adoption of non-neutral postures during SP use may also contribute to musculoskeletal disorders. In the contemporary scenario, the present study aimed to assess the association between SP usage and musculoskeletal outcomes among medical students.

Methods

The phase II MBBS students were stratified into low (score of ≤30) and high (score of ≥30) users based on their response to the SAS-SV (Smartphone Addiction Scale-Short Version). The objectives were: (i) comparing forward head protrusion (FHP), measured using the craniovertebral angle (CVA); (ii) documenting the region of maximum pain among the neck, hands, and joints of both upper limbs using the visual analogue scale (VAS); and (iii) assessing the cross-sectional area (CSA) and circumference of the median nerve (MN) within the carpal tunnel, as well as the CSA of the flexor pollicis longus (FPL) tendon, measured at the mid-thenar level using ultrasonography.

Results

The CVAs of the low and high users were 45.89 ± 5.8 and 45.24 ± 4.8, respectively. The lower the CVA, the higher the FHP. The prevalence of neck pain was statistically higher among high users (p < 0.05). Pain was reported more frequently among high users in the shoulder and thenar regions, but this was not statistically significant. The circumference (1.10 ± 0.15), CSA (0.09 ± 0.16) of the MN, and thickness of the FPL (0.14 ± 0.27) were increased in high users in their dominant (right) hand compared to low users (1.08 ± 0.14, 0.06 ± 0.01, and 0.11 ± 0.19, respectively).

Conclusion

The study documents a possible association between increased SP use and musculoskeletal discomfort, particularly in the neck, but objective structural changes were not evident. The findings were not statistically significant, which may partly be due to methodological limitations. The planning and development of more specific preventive measures, which are critical for reducing SP-associated repetitive strain injuries, would benefit from future studies incorporating larger samples, dynamic assessments, and better control of confounding factors to clarify these relationships.

## Introduction

With the revolution in technology, digital devices have become integral to daily life, and life is gradually becoming difficult without their presence. Among all others, smartphone (SP) usage shows a rapid surge, abrogating the frontiers. Statistics in India show that in early 2024, there were 1.12 billion active SP users, which is equivalent to 78% of the country’s population [1]. There is growing concern about SP overuse. The gamut of disorders ranges from defective cognition among children, sleep disturbance, musculoskeletal, and behavioural disorders [2,3].

The altered neck and body posture the users adopt abysmally impacts even respiratory function [4]. The positive correlation between SP usage and neck pain is evident [5]. SP tucks the human hand busy in various ranges of movements, causing excessive mechanical strain on digits, especially the thumb [6]. The weird posture of users abets musculoskeletal disorders in the neck and upper back.

Blinded by the positivity of technological advancement that kills time, space, and distance, mankind fails to contemplate the metamorphosis occurring in various body regions. Addiction-like behaviours associated with SP usage were observed among Indian medical graduates [7]. Work-related musculoskeletal disorders of the neck, upper extremities, and wrist frequently occur due to increased usage of computers [8,9].

Currently, computers are being replaced by SPs in the major arena, and it is obvious to expect various effects of this. However, despite the increasing use of SPs, there is limited evidence on objective morphological changes, particularly involving soft tissue structures such as tendons and nerves. The joints of the thumb and thenar muscles, especially the flexor pollicis longus (FPL) tendon and the median nerve (MN), which innervates the same, need special observation.

With the contemporary scenario, this study aimed to evaluate the association between SP usage and musculoskeletal outcomes among medical students. The primary objectives were to compare forward head posture using craniovertebral angle (CVA) and assess pain distribution and severity using a visual analogue scale (VAS) between low and high SP users. Secondary objectives included evaluating morphological changes in the MN and FPL tendon using ultrasonography.

## Materials and methods

Study design

This will be a comparative cross-sectional study. The objectives of the study are to assess the extent of usage of SPs among the study participants and to compare the documented morphological parameters, discussed below, with the level of usage of SPs.

Study population and sample size

The study population belongs to phase II MBBS students who gave informed consent to participate in the study. A sample size of 54 was calculated based on previous study results using OpenEpi version 3.0, considering a 95% confidence level, 80% power, and the expected effect size derived from earlier literature [4].

Inclusion and exclusion criteria

The inclusion criteria are medical students of third to fifth semesters. The exclusion criteria are medical students with H/O arthritis, cervical spondylosis, spondylolisthesis, or any other bone disease or injury, and students using other personal digital assistants (PDAs) for gaming, animation, etc.

Data collection procedure

The study was started after obtaining Institutional Ethics Committee clearance. The Smartphone Addiction Scale-Short Version (SAS-SV) questionnaire was distributed to all voluntary participants [10]. The questionnaire had 10 questions with a six-point Likert scale. Based on the scores, the respondents were stratified into two groups: low SP users (score ≤ 30) and high SP users (score ≥ 30). So, a total of 54 healthy medical students (29 females and 25 males), with 27 from each group, were included. Participants were selected using a systematic approach based on roll numbers, every third student, which provided a practical and evenly distributed sampling framework across the batch while minimising clustering of participants. This method was adopted for feasibility within an academic setting and to ensure representation across the group. The average duration of SP use was lower in the low-user group (approximately four hours per day) and higher in the high-user group (approximately seven hours per day). All participants were right-handed and reported using both hands while operating their SPs. SP use was reported across different postures, including standing, sitting, and lying positions.

**Figure 1:**
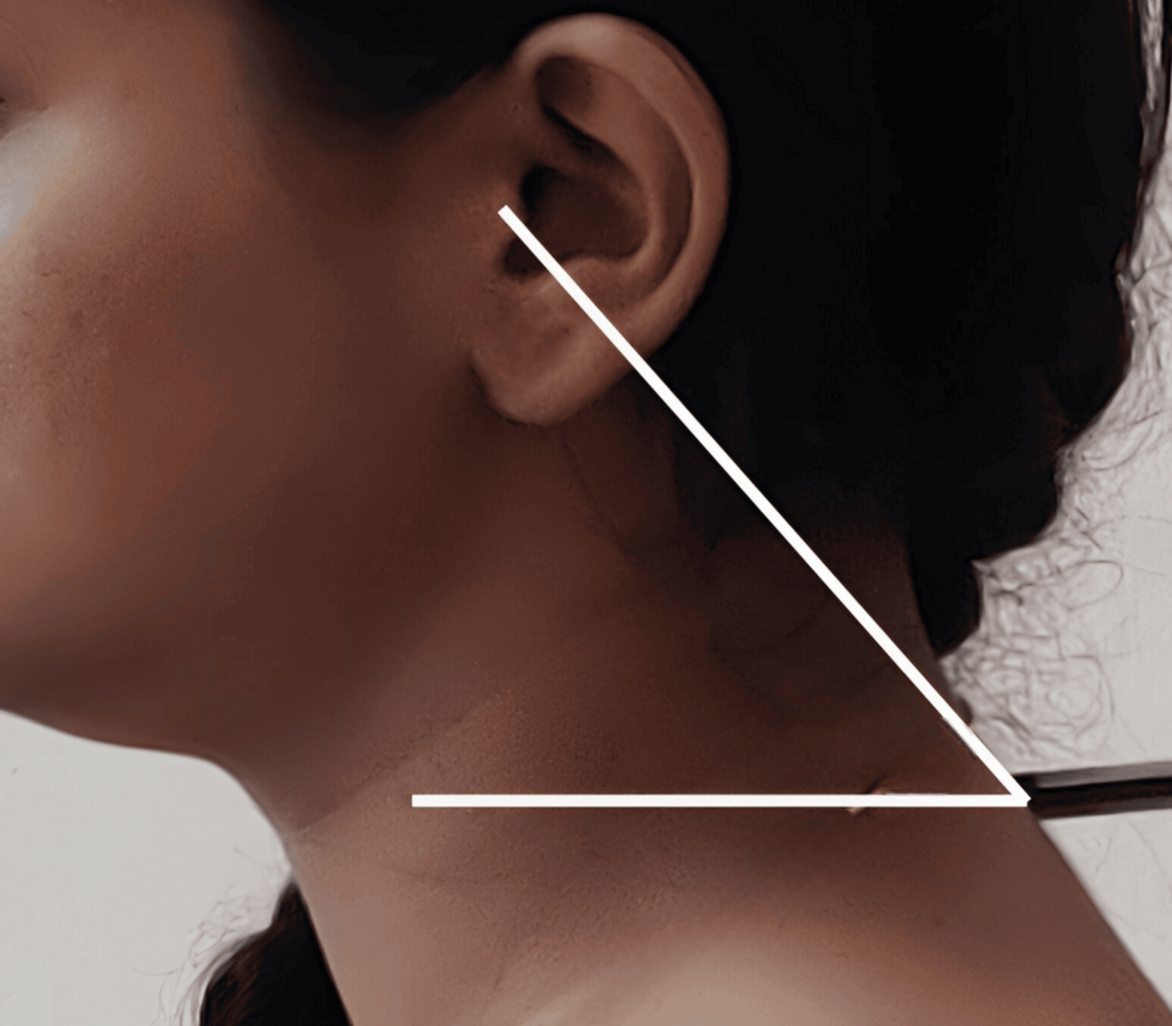
Lines for measurement of CVA C7 point, horizontal line through C7, and a line connecting C7 to the tragus. CVA: craniovertebral angle

**Figure 2:**
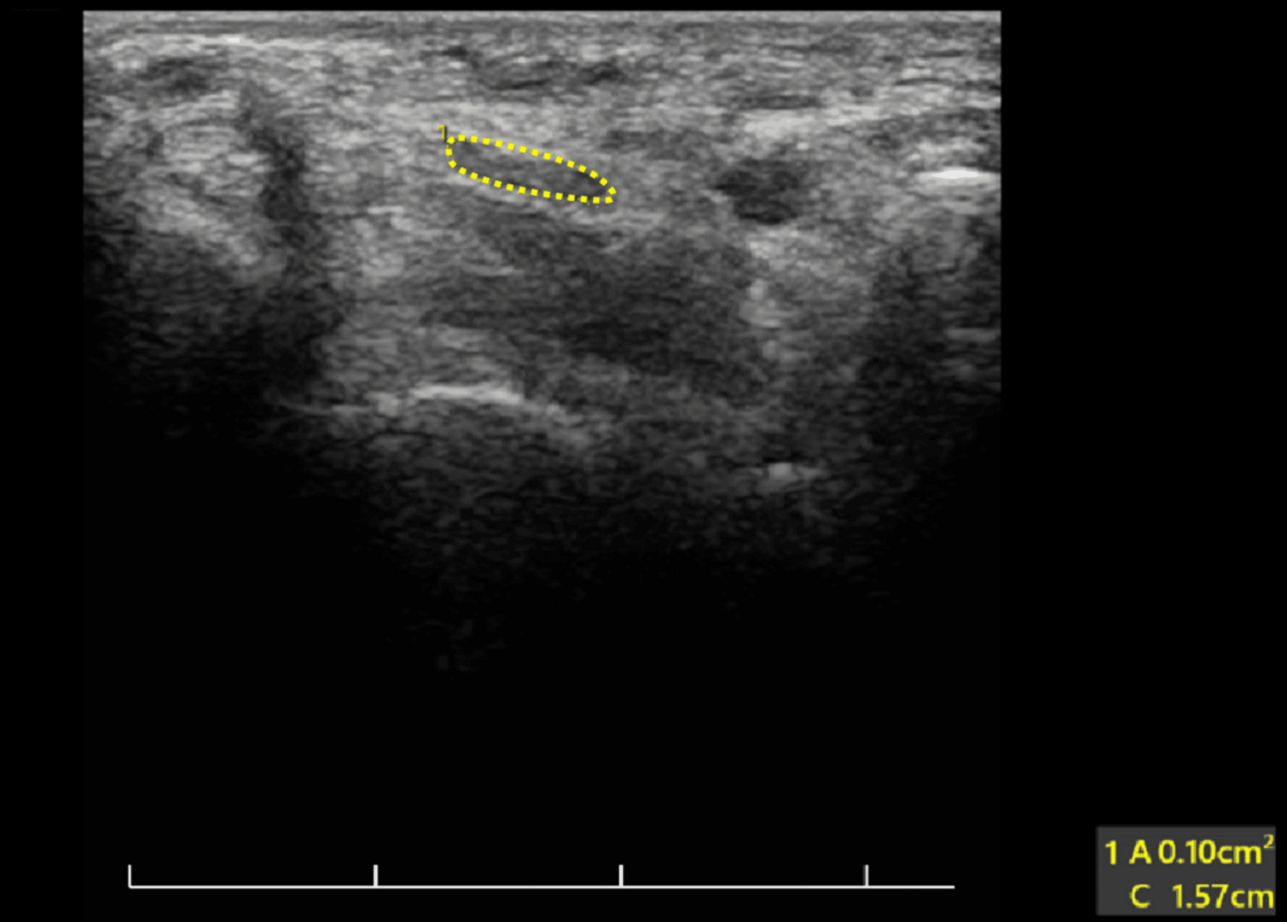
USG showing a cross-section of the median nerve in the carpal tunnel of the left hand of a low user (dotted yellow lines) USG: ultrasonography

CVA was measured to assess forward head protrusion (FHP). The lower the CVA, the higher the FHP. This was done using a digital lateral-view photograph of participants in a normal standing position, and the angle was measured digitally using Adobe Photoshop CS6 software (Adobe Systems Inc., San Jose, CA, USA). Measurements were performed by an independent assessor trained in the use of Adobe Photoshop CS6 and blinded to participant details. Each measurement was obtained three times at different time intervals, and the mean of the three readings was used for analysis to minimise intra-observer variability. To ensure uniformity, the camera was mounted on a fixed tripod and positioned perpendicular to the horizontal plane. The spinous process of the seventh cervical vertebra was marked with a pointer. CVA, which is the angle between the horizontal plane passing through C7 and a tangential line connecting C7 with the tragus, was measured digitally using Adobe Photoshop CS6 (Figure 1) [11]. The photographic method for measuring CVA was adopted as it showed good validity and reliability in previous studies (intra-class correlation coefficients ranged from 0.88 to 0.98) [12]. A VAS was used to assess pain in the neck, hands, and joints of both upper limbs. The users categorised the region of maximum pain. Bilateral ultrasonographic assessment was performed using a Philips HD7 system (Philips Healthcare division, Amsterdam, Netherlands) with a 12 MHz linear probe by a sonologist who was blinded to the participant’s group allocation. Measurements were obtained with participants in a comfortable supine position, with the forearm fully supinated and the hand resting on a support. Axial images were used to assess the cross-sectional area (CSA) and circumference of the MN within the carpal tunnel (Figure 2), as well as the CSA of the FPL tendon at the mid-thenar level. Each parameter was measured three times, and the mean of the three readings was used for analysis.

Statistical analysis

Categorical variables have been described in counts and percentages. Continuous variables are described by mean and standard deviation. Normality of the data was analysed using the Shapiro-Wilk test. The Shapiro-Wilk statistic was more than 0.91 for all the variables, and the p-value was not significant, denoting a normal distribution of the data. So, Student’s unpaired t-test was used to compare the morphological parameters between low and high users. Categorical variables were compared using the Chi-square test.

## Results

Demographic details of the participants are described in Table 1. The mean age (in years) and gender distribution were almost equally distributed among the low- and high-user groups. The mean age in the low group was 18.2 years, and in the high group was 18.3 years. There were 12 and 13 males in the low and high groups, while 15 and 14 females were there in the low and high groups, respectively.

**Table 1:** Demographic details

Participants	Low	High
Mean age (years)	18.2	18.3
Hours of usage	4	7
Male (n = 25)	12	13
Female (n = 29)	15	14
Total (n = 54)	27	27
Handedness	Right dominant	Right dominant
Hand usage	Both hands	Both hands

The mean CVA in the neutral position among the low-user group was 45.89°, and in the high-user group it was 45.24°. The difference in the angle between the groups was not statistically significant, as mentioned in Table 2.

**Table 2:** Comparison of the mean craniovertebral angle (CVA) in the neutral standing position

Parameter	Low user	High user	p-value
CVA	45.89 ± 5.8	45.24 ± 4.8	0.7

Region and proportion of pain scores among the users are described in Tables 3-4. The mean pain scores were comparatively higher among the high-user group in the regions of neck, thenar region, shoulder, and elbow. Among low users, neck pain was found to be higher, with 43% of users reporting it, whereas 39% of users reported pain in the thenar region, 21% in the shoulder, and 18% in the elbow.

**Table 3:** Region and proportion of pain among users

Region of pain	Low user (%)	High user (%)	p-value
Neck	43	79	0.018
Thenar	39	62	0.18
Shoulder	21	45	0.11
Elbow	18	23	1.00

**Table 4:** Proportion of pain severity scores using the visual analogue scale (VAS) in both upper limbs None of the p-values was significant between the users.

Parameters	Users (N = 54) (low n = 27 & high n = 27)	No pain (VAS = 0) (%)	Mild to moderate (VAS = 1-6) (%)	Severe (VAS = 7-10)
R thenar	Low	85.2	14.8	-
	High	59.3	40.7	-
L thenar	Low	96.3	3.7	-
	High	88.9	11.1	-
R Elbow	Low	70.4	29.6	-
	High	63	37	-
L elbow	Low	92.6	7.4	-
	High	81.5	18.5	-
R shoulder	Low	77.8	18.5	3.7
	High	70.4	25.9	3.7
L shoulder	Low	89	11	0
	High	74	26	0
R neck	Low	59	37	3.7
	High	44.5	48.1	7.4
L neck	Low	77.8	18.5	3.7
	High	51.9	44.4	3.7

The proportion of pain in various regions of high users was similarly distributed across regions as in low users, but at a higher frequency. The frequency of users reporting pain was 79% in the neck, 62% in the thenar region, 45% in the shoulder region, and 23% in the elbow region. Considering the severity of pain, severe pain was perceived by both user groups in the regions of the right shoulder and right and left sides of the neck. None of the participants reported severe pain in the left shoulder compared to the right, which indicates that dominant hand usage is associated with pain. There was a statistically significant difference in the prevalence of neck pain between low and high SP users (p = 0.018). However, differences observed in the thenar, shoulder, and elbow regions were not statistically significant (p > 0.05).

The CSA and circumference of the MN, as well as the CSA of the FPL muscle at the mid-thenar level, were measured in both the right and left hands (Table 5). All comparisons showed p-values > 0.05, indicating that there were no statistically significant differences between the groups. In the right hand, the mean CSA of the MN in the high-user group (0.09 ± 0.16 mm²) was greater than in the low-user group (0.06 ± 0.01 mm²) (p = 0.43). Similarly, the mean circumference of the MN was higher in the high-user group (1.10 ± 0.15 cm) compared to the low-user group (0.99 ± 0.27 cm) (p = 0.08). The mean CSA of the FPL muscle also increased in the high-user group (0.14 ± 0.27 mm²) versus the low-user group (0.06 ± 0.01 mm²) (p = 0.13).

**Table 5:** USG of the median nerve and FPL tendon USG: ultrasonography; CSA: cross-sectional area; FPL: flexor pollicis longus

Parameters	Right hand (mean ± SD)			Left hand (mean ± SD)		
	Low	High	p-value	Low	High	p-value
CSA of the median nerve (mm^2^)	0.06 ± 0.01	0.09 ± 0.16	0.43	0.05 ± 0.01	0.06 ± 0.01	0.60
Circumference of the median nerve (cm)	0.99 ± 0.27	1.10 ± 0.15	0.08	1.01 ± 0.21	1.08 ± 0.14	0.19
FPL at mid-thenar level (mm^2^)	0.06 ± 0.01	0.14 ± 0.27	0.13	0.06 ± 0.01	0.11 ± 0.19	0.20

In the left hand, the mean CSA of the MN was similar between the high (0.06 ± 0.01 mm²) and low (0.05 ± 0.01 mm²) groups (p = 0.60). The MN circumference in the left hand was slightly higher in the high-user group (1.08 ± 0.14 cm) compared to the low-user group (1.01 ± 0.21 cm) (p = 0.19). The CSA of the FPL muscle in the high (0.11 ± 0.19 mm²) and low (0.06 ± 0.01 mm²) user groups showed no significant difference (p = 0.20).

## Discussion

The present study attempted to identify low and high users of SP and studied the correlation between the CVA in neutral position, pain in the neck and upper limb, and observable changes in the structures in the carpal tunnel among low and high SP-using healthy college students. The SAS-SV was used to identify low and high SP users. Pain was documented using a VAS, and morphometry of structures in the carpal tunnel was assessed using USG among SP users, irrespective of the degree of usage. The findings showed no significant variation in the CVA and morphometry. Although higher reporting of subjective pain in the neck, thenar, and shoulder regions was observed among high SP users compared to low SP users in the dominant hand, these differences were statistically significant only in the overall prevalence of pain in the neck region.

When an individual uses an SP, he/she tends to flex the neck, and if this posture, i.e., forward head posture, is adopted for a prolonged duration, it causes pain and discomfort in the neck region [13]. This compensatory posture is associated with neck bending, increasing neck muscle activity. A study conducted among 100 South Korean adolescents demonstrated a significant flexed posture among SP-addicted participants [9]. In the present study, we could not find a significant difference between the two groups concerning the CVA. This is similar to the study by Grob et al. [14] and Kumagai et al. [15], which found no association between global cervical curvature and neck pain in adults with text neck syndrome. In contrast, a study conducted in Hong Kong among 560 students demonstrated an association between increased CVAs and musculoskeletal pain in high SP users [16]. Hence, we can perceive that the magnitude of SP usage need not necessarily be associated with the change in the CVA. One reason that can be attributed to this finding is that the study involved young participants, in whom the resilience and resistance of the spinal ligaments, including cervical vertebrae, are strong enough to resist structural deformation compared to older age groups [17]. So, whenever the load is applied during SP usage, the postural stress on the neck returns to normal once the load is removed. Therefore, no significant correlation was observed in the CVA between the users. Also, the time taken for observable changes to manifest varies, and this could not be documented unless the participants are followed up with for a prolonged period.

In our study, we observed that high-SP users had a higher likelihood of experiencing pain in the neck, thenar, and shoulder region than their counterparts. This is similar to the study by Mongkonkansai et al. [18], where students using SPs for more than 60 minutes per day exhibited a ten times higher likelihood of having musculoskeletal disorders than those using them for less than 60 minutes daily. Taking insights from the studies published by Joergensen et al. and Paananen et al. [19,20], it can be postulated that being plastered to the SP, especially in an improper ergonomic posture, is one of the highly prevalent reasons for musculoskeletal discomfort and fatigue among adolescents. Though we could not ascertain the position in which the participants used SPs in the past, this increased prevalence of musculoskeletal discomfort can be attributed to decreased endurance of the relevant musculature [21]. The correlation between high SP usage and thumb pain is similar to that reported in studies conducted in Brazil and Saudi Arabia [22,23]. This could be due to prolonged holding of the SP between the thumb and other fingers.

Interestingly, when we tried to validate self-perceived pain objectively using the VAS, we could not demonstrate statistically significant variation between the two groups across different muscle groups. Considering the commonly adopted posture, which puts extra stress on the extensor muscles of the neck [24] and the upper trapezius - which demonstrates the highest fatigability at a 50° angular position [25] - it could be postulated that prolonged and high usage of SPs can create a sense of discomfort that is subjectively perceived but not objectively demonstrable [26]. However, it needs to be noted that, being a cross-sectional study and considering the age of the participants, they might have had a corrective counter-mechanism, which would have masked clinical findings at the time of assessment. With decreasing endurance with age and other comorbidities, obvious pathologies related to the neck might manifest. Hence, they need to practise neck muscle and upper trapezius strengthening exercises. Another possible reason may be the long study hours and static neck posture adopted by medical students, which can cause stress to neck muscles and eventually pain. So, this may have masked the effect of SP use on neck pain when measured [27].

The final component of the study aimed to compare the CSA, circumference of the MN, and FPL between high and low SP users. In our study, we could not appreciate significant differences in the CSA between the two groups. Though there were non-significant differences in the circumference of the MN and FPL between both groups, these could not be attributed to SP usage per se. In our study, none of the participants had objectively demonstrable carpal tunnel syndrome (CTS), and measurements were taken in the resting position, where the students were not asked to perform any forceful pinch grip equivalent to holding the SP and typing on it. Previous studies [28,29] have demonstrated an increase in the CSA in high-intensity users, and their theories posit that whenever the MN is unable to glide in the carpal tunnel, there is a greater chance of nerve compression, which could especially be observed during pinch grip [30]. In another study by Woo et al. [31], intensive users had larger MN CSAs, flattening ratios, and perimeters than their counterparts. Also, the CSAs were decreased under finger flexion and grip, wrist extension-flexion, and radial-ulnar deviation compared to the neutral wrist position. Similarly, another study [32] showed that individuals who used SPs for more than four hours per day had a significantly higher association with CTS, and those who held SPs with both hands had 7.8 times higher odds of developing CTS than those who used one hand. In yet another study, the CSA of the MN in the dominant hand was significantly increased in SP users, irrespective of usage magnitude [33].

Similarly, we could not observe any significant morphological changes in the CSA of the FPL among the two cohorts. This could be because most of our participants, who were millennial learners, had the habit of using both hands for typing. In their study, Xie et al. [34] observed an increase in electromyographic activity in the upper limbs of those who had the habit of typing with only one hand. However, if both hands are used for typing, the force would be dissipated and hence may not result in significant morphological changes related to the FPL. Also, for morphological changes to be manifested, the mechanical load should be strong enough and sustained for a longer duration rather than short repetitive lower loads, which occur during SP use [35].

Limitations

Being a cross-sectional study that also included participants from similar backgrounds, the present study could not establish causal relationships with sufficient strength. The relatively small sample size may have further reduced the ability to detect subtle differences. Also, the results cannot be generalised to a larger population at the present stage. The study used a systematic sampling approach based on roll numbers, which may not provide the same level of randomness as true random sampling. Although this method allowed for a structured and feasible selection of participants, the possibility of selection bias cannot be completely excluded. Future studies using fully randomised sampling methods would help improve representativeness. The overall classification was made based on the SAS questionnaire. We also need to consider recall bias in participants while answering the questionnaire, which could compromise the study’s strength. The study was conducted during the post-COVID period, when the duration of usage increased in both groups. Also, potential confounding factors such as posture habits, duration and pattern of SP use, and levels of physical activity were not specifically controlled or quantified. These factors may have influenced the observed outcomes and should be considered while interpreting the results. Ultrasonography measurements were done in the resting position, and dynamic assessment was not performed. As a result, changes that might occur during movement or normal physiological activity could not be evaluated. Age, gender, and hours of usage may act as possible influencing factors; therefore, the independent association between SP use and the outcome variables could not be fully assessed. Given these limitations, the findings should be interpreted with caution, and further studies with larger sample sizes, randomised sampling methods, prospective designs, and incorporation of objective and standardised assessment of exposure and confounding variables are recommended to improve validity and reproducibility.

## Conclusions

In this study, a statistically significant difference between the groups was observed only in the prevalence of neck pain. Although shoulder and thenar discomfort were reported more frequently among high SP users, these differences did not reach statistical significance. Additionally, no significant differences were identified in objective measures, including the cross-sectional area of the MN and the FPL tendon. Overall, while the findings suggest a possible association between increased SP use and musculoskeletal discomfort, particularly in the neck, objective structural changes were not evident. Further well-designed studies incorporating larger samples, dynamic assessments, and better control of confounding factors are needed to clarify these relationships and support the development of targeted preventive strategies for SP-related repetitive strain injuries.
